# An activity theory perspective of how scenario-based simulations support learning: a descriptive analysis

**DOI:** 10.1186/s41077-017-0055-0

**Published:** 2017-11-21

**Authors:** Alexis Battista

**Affiliations:** Uniformed Services University of the Health Sciences, Graduate Programs in Health Professions Education, Department of Medicine, F. Edward Hébert School of Medicine, 4301 Jones Bridge Road, Bethesda, MD 20814-4712 USA

**Keywords:** Activity theory, Distributed cognition, Legitimate peripheral participation, Scenario-based simulation, Simulation, Simulation-based instructional design, Simulation-based learning

## Abstract

**Background:**

The dominant frameworks for describing how simulations support learning emphasize increasing access to structured practice and the provision of feedback which are commonly associated with skills-based simulations. By contrast, studies examining student participants’ experiences *during* scenario-based simulations suggest that learning may also occur through participation. However, studies directly examining student participation during scenario-based simulations are limited. This study examined the types of activities student participants engaged in during scenario-based simulations and then analyzed their patterns of activity to consider how participation may support learning.

**Methods:**

Drawing from Engeström’s first-, second-, and third-generation activity systems analysis, an in-depth descriptive analysis was conducted. The study drew from multiple qualitative methods, namely narrative, video, and activity systems analysis, to examine student participants’ activities and interaction patterns across four video-recorded simulations depicting common motivations for using scenario-based simulations (e.g., communication, critical patient management).

**Results:**

The activity systems analysis revealed that student participants’ activities encompassed three clinically relevant categories, including (a) use of physical clinical tools and artifacts, (b) social interactions, and (c) performance of structured interventions. Role assignment influenced participants’ activities and the complexity of their engagement. Importantly, participants *made sense* of the clinical situation presented in the scenario by reflexively linking these three activities together. Specifically, student participants performed structured interventions, relying upon the use of physical tools, clinical artifacts, and social interactions together with interactions between students, standardized patients, and other simulated participants to achieve their goals. When multiple student participants were present, such as in a team-based scenario, they distributed the workload to achieve their goals.

**Conclusion:**

The findings suggest that student participants learned *as they engaged* in these scenario-based simulations when they worked to make sense of the patient’s clinical presentation. The findings may provide insight into *how* student participants’ meaning-making efforts are mediated by the cultural artifacts (e.g., physical clinical tools) they access, the social interactions they engage in, the structured interventions they perform, and the roles they are assigned. The findings also highlight the complex and emergent properties of scenario-based simulations as well as how activities are nested. Implications for learning, instructional design, and assessment are discussed.

## Background

### The need to examine differences in scenario-based simulation contexts

The dominant frameworks for describing how simulations support learning emphasize increasing access to structured practice (i.e., repeated or deliberate practice) and the provision of feedback [1, 2]. However, our understanding of how simulations support learning (e.g., structured practice, feedback) may be influenced by learning strategies employed in skills-based simulations which prioritize focused practice of a singular skill, while scenario-based simulations present student participants with a holistic problem or situation to analyze. For example, in Issenberg and colleagues’ Best Evidence Medical Education (BEME) Review, of the 109 included studies, 90 (83%) examined skills-based contexts [1]. Additionally, McGaghie and colleagues’ meta-analysis compared simulation-based medical education (SBME) using deliberate practice (DP) with learning in the clinical setting; of 14 included studies, 12 (86%) addressed learning in skills-based simulation contexts versus scenarios [2].

However, although both skills- and scenario-based simulations are commonly employed, scenario-based simulations (ScBS) may have unique properties in design and implementation that set them apart which, in turn, may influence learning processes. For example, skills-based simulations primarily emphasize the focused teaching and practice of a specific procedural skill [3, 4]. By comparison, ScBS are often employed when the desired learning outcomes include working in a team-based context, practicing communication skills, or responding to a crisis or critical patient event [5]. Additionally, whereas skills-based simulations often seek to minimize complexity, ScBS are employed to incorporate the complexities associated with clinical practice, including engaging socially with the patient or support persons (e.g., simulator, standardized patient) and interacting with other healthcare professionals [5–7].

ScBS are also described as possessing characteristics associated with sociocultural and situated learning practices. For example, in a ScBS, a narrative is employed to guide student participants’ engagement in which learning activities are designed around a story or a problem that needs to be explored or resolved [6]. Furthermore, in a ScBS, student participants are assigned to designated clinical roles, such as that of the nurse, physician, or other healthcare professionals, and are expected to conform to the behavior- and practice-oriented conventions of their assigned roles [5, 8, 9]. However, despite these differences in use, design, and implementation, simulation-based learning (SBL) research rarely disaggregates differences between skills-based and scenario-based simulations.

### The need to examine how participation during ScBS supports learning

To date, empirical studies in health professions simulation have frequently focused on determining *if* SBL supports improvements in participant satisfaction, diverse learning outcomes, and in some cases, outcomes in the clinical setting. For example, several meta-analyses suggest that SBL supports improvements in medical knowledge, psychomotor and communication skills, time to complete a skill, and self-efficacy [2, 10, 11].

In addition, learning associated with scenario-based simulations often prioritizes reflection and debriefing to support learning [1, 2, 12, 13]. For example, according to Rudolph and colleagues, reflection helps individuals make sense of their experiences and scrutinize their assumptions and beliefs [14]. Palaganas and associates suggest that student participants can also engage in sense-making efforts through discussion with faculty and peers when they pause to reflect during a scenario [13]. Furthermore, according to Fanning and Gaba, participation in a simulated encounter is often considered a normalizing event in which student participants engage in a shared experience (e.g., code team response) that, in turn, enables reflection processes [12]. Although reflection is critical to learning, these perspectives primarily emphasize the role that verbal discourse can play in learning processes, yet studies examining how student participants may learn through participation in a ScBS are limited. Furthermore, framing learning as an activity that occurs after a simulation reflects traditional cognitive perspectives of learning.

By contrast, some studies examining student participants’ experiences in simulation suggest they may learn *during* scenario participation. For example, Kneebone and colleagues assigned student participants to one of two procedural scenarios: insertion of a urinary catheter or wound closure using a hybrid simulation strategy [3]. Student participants reported that in addition to learning the designated skill, they also became aware of the importance of maintaining patient privacy, learning where to find supplies and materials, and interacting with the standardized patient [3].

In another study, Mikkelsen and colleagues assigned student participants to either case study sessions or a ScBS to learn how to manage cross infections [15]. Student participants who engaged in the ScBS reported that as they partook in the scenario, they became aware of the complexities of patient care through the consequences of their actions. For example, if a student participant did not acquire the proper equipment or supplies before entering the room of a patient on isolation precautions, they then had to exit, collect the needed supplies, and start again [15]. As a result of participation, student participants not only learned how to care for a patient on isolation precautions, but also learned the importance of pre-planning their care.

Furthermore, Lasater [16] examined student participants’ experiences of learning clinical judgment using ScBSs. The findings suggested that student participants learned during the ScBS when the simulator physiologically responded to their actions, thus allowing them to understand the consequences of their actions (e.g., cardiopulmonary depressions following administration of a narcotic agent) [16].

Importantly, the findings from Lasater, Mikkelsen et al., and Kneebone et al. suggest that student participants learned through the process of participation in a ScBS [3, 15, 16]. Although these studies importantly focused on student participants’ self-reported experiences, there have been no studies focusing on which activities could promote learning within a ScBS. One of the first steps is to undertake a comprehensive examination of participant activity to understand how ScBSs may support learning.

### The current study

The following three features were taken into account when considering how ScBS may support learning: the dominant frameworks for describing how ScBS support learning emphasize increasing access to structured practice and the provision of feedback; studies examining student participants’ experiences during ScBS participation suggest learning may occur through participation; and that the direct examination of participant activity is limited. The purpose of this study was to examine the types of activities student participants engaged in and then analyze their patterns of activity to consider how ScBS participation may support learning. The aims of this study were (1) to make explicit the types of activities student participants engage in and (2) to examine *how* student participants’ activities during participation in scenario-based simulations may contribute to their learning. The research questions were:

1. What types of activities do student participants engage in during participation in scenario-based simulations?
  - What is the frequency and regularity of these activities across different scenario types?
2. How do student participants engage in scenario-based simulations (e.g., how do they organize their activities, interact with each other)?

## Methods

### Theoretical framework: activity theory

To support this study’s goal of in-depth analysis of participant activity, this study was informed by Engeström’s first-, second-, and third-generation perspectives on activity theory (AT). AT is often used by researchers as a descriptive tool to map the interactions between individuals and their environment [17, 18]. Systematically mapping activity can help researchers examine meaning-making processes as embedded in dynamic emergent systems [17, 19].

According to Engeström, first-generation AT emphasizes Vygotsky’s mediated action triangle that highlights how individuals use artifacts or tools to achieve their goals [17]. Engeström’s second-generation AT, often referred to as activity systems analysis, advances first-generation perspectives because it helps elucidate the collective nature of human activity by accounting for the roles individuals play and the rules that may guide them [17]. This added emphasis on roles and rules is intended to highlight the complex interactions within an activity system [17, 19]. Third-generation AT supports analysis of multiple points of view to highlight interactions between activity systems [18, 19]. The use of third-generation AT requires the analysis of at least two activity systems, though multiple activity systems may be available for examination [18].

Central to all three generations of AT is the inseparability of learning and doing. According to AT, learning is conceptualized as practice [18]. This contrasts with traditional learning theories that position learning as occurring prior to or after an individual’s performance in an activity. Importantly, when discussing activity, activity theorists are not just concerned with what individuals do, but are also interested in *doing* as a transformative process [17, 20]. For example, nursing students participating in a ScBS often must assess a patient’s vital signs. In assessing vital signs, students may perform specific activities, such as palpating a pulse or using a blood pressure device. The practice of assessing vital signs is governed by specific rules, such as how and where to place the blood pressure cuff or where to locate the pulse. In performing these activities, nursing students gain new knowledge about the patient that they must then integrate into their care plans. They must consider the information gained, decide what it may mean (e.g., hemodynamically stable or unstable), and, in turn, determine what they should do next.

**Fig. 1:**
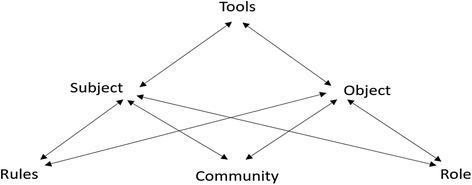
Activity system diagram. The figure illustrates the mediated relationship between subjects and tools and the interrelationships among role assignment and rules and conventions of participation [17–19]

Yamagata-Lynch defines activities as “mediational processes in which individuals and groups of individuals participate, driven by their goals and motives, which may lead them to use new artifacts or cultural tools” (p. 17) [17]. Participants can include an individual or a group of people engaged in an activity [21]. Their goals or objectives are the physical or mental product(s) they seek during participation (e.g., postpartum assessment) [21]. *Tools* include culturally specific artifacts that participants use to achieve their goals [21]. For example, when a participant assesses a patient’s vital signs, they may use a manual or automated blood pressure device to achieve this goal. *Rules* are the guidelines, conventions, or protocols that govern participants’ activity in the system, either explicitly or implicitly, such as patient care guidelines or commonly accepted assessment processes [21]. *Roles* are the division of labor among actors in the system [21]. Figure 1 provides a generic AT diagram which is often used to heuristically represent AT concepts.

### Design

An exploratory descriptive analysis was selected using multiple qualitative methods, namely, narrative, video, and activity system analysis, to support the goal of rich description of the types of activities student participants engaged in that may support learning. The strategy of using multiple qualitative methods was selected to help better account for the complex and dynamic activity usually present in a ScBS.

### Video sample

A purposive sample of four video-recorded scenario-based simulations representing commonly cited motivations for employing them (e.g., team training, communication skills) was sought [22]. Additional sampling criteria included (a) ScBS that were designed to represent the complexity and the social practices of caring for patients and (b) inclusion of student participants who regularly engaged in ScBSs, which was reasoned would afford analysis of student participants’ typical activity, rather than the activity of student participants becoming familiar with engaging in a ScBS.

Following institutional review board (IRB) approval, 34 previously video-recorded scenarios were identified. Because the identified video recordings were captured during the National Council of State Boards of Nursing (NCSBN) National Simulation Study, approval from the NCSBN was also sought. The identified videos had been recorded during the third semester of an accelerated second-degree baccalaureate program during a Nursing the Childbearing Family course. All student participants portrayed in the videos had participated in ScBS activities at least every other week, so they had the requisite experience that was sought.

Following discovery, all videos and accompanying instructional design documents were reviewed to assess quality [23]. Of 34 videos, 19 were eliminated because the videos were incomplete, had poor sound quality, or could not be transformed for video analysis. The 15 remaining videos were categorized by scenario name, scenario emphasis, nursing student participant roles, patient and clinical roles, and length. Following this step, four high-quality videos were selected for analysis, making sure to include videos capturing common motivations for using ScBSs, namely a non-emergent patient assessment, an urgent patient presentation, a team-based scenario, and communication skills. Table 1 summarizes the videos selected for in-depth analysis.

**Table 1:** Summary of selected video-recorded scenario-based simulations and their characteristics

Scenario name	Faculty scenario goal	Roles portrayed by student participants	Roles portrayed by standardized patients and other simulated participants	Scenario duration (in minutes)
Uncomplicated postpartum assessment	Conduct and uncomplicated postpartum assessment	Primary nurse Support nurse	Noelle	9:06
Post-epidural hypotension	Escalate care and treat a patient experiencing post-epidural hypotension	Primary nurse Support nurse	Noelle Patient’s mother Charge nurse Anesthesiologist	15:34
Postpartum hemorrhage	Recognize and treat a patient experiencing a postpartum hemorrhage	Primary nurse Support nurse 1 Support nurse 2	Noelle Patient’s spouse Charge nurse Obstetrician	11:01
Fetal demise	Conduct an intrapartum assessment of a patient experiencing a fetal demise in utero	1 primary nurse	Standardized patient Patient’s spouse	29:04

*Noelle* embodied the patient while a standardized patient portrayed her voice to support verbal social interactions

### Analysis

Data analysis was conducted in multiple stages. Stage one focused on identifying the types of activities in which student participants engaged in and verifying their frequency and regularity across all four videos. Stage two focused on re-aggregating the activity system to support analysis of the interactions between student participants and the ScBS context, which included the physical environment, standardized patients, and other simulated participants, for example [17].

### Stage one

Drawing on Ollerenshaw and Creswell’s descriptive restorying methods [24], a narrative describing each participant’s activities was composed [25]. First- and second-generation activity theories were used to help focus restorying (e.g., recording what tools nursing students used, who they communicated with, verbalized goals). Additionally, except for ahs, uhms, and pauses, each video was transcribed verbatim, including the social utterances of all participants (e.g., student participants, standardized patients, and other simulated participants). Lastly, each narrative and transcription was re-reviewed for accuracy. This first stage served to disaggregate the rich and dense activities of the scenario.

Following restorying, open coding procedures were used to categorize student participants’ activities. This entailed reading and coding each restoryed narrative and transcript to identify the tools, roles, and goals of each participant [26, 27]. The instructional design documents for each scenario were also read to search for additional tools, roles, and scenario goals.

Initial codes were then compared across the four videos using video analysis software (Studiocode) to determine their regularity and frequency. Accounting for regularity and frequency helped verify initial codes and identify new codes that were not revealed during earlier coding efforts. Following this step, initial codes were consolidated into three major categories by grouping similar initial codes together.

### Stage two

**Fig. 2:**
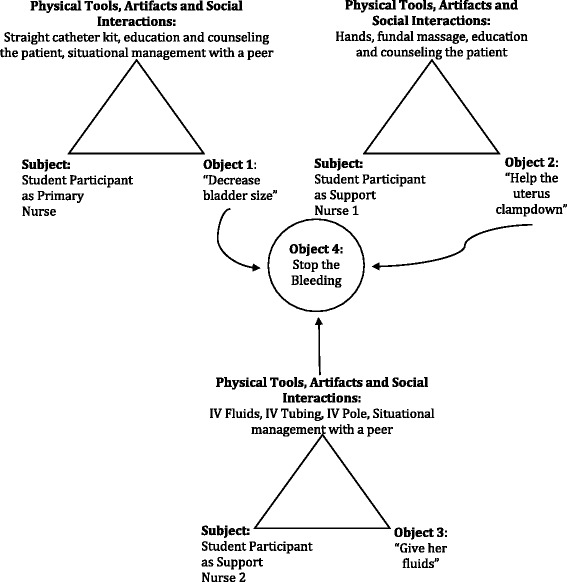
Coordinating and distribution of activities of postpartum hemorrhage. This diagram demonstrates how three student participants distributed their activities and goals to achieve the larger goal of treatment of the patient’s postpartum hemorrhage and hypotension. Goals (objects) reflect participants’ utterances during the scenario

During the second stage of analysis, which supported the goal of analyzing *how* student participants engaged, a second cycle of coding was conducted [26]. This involved drawing on second- and third-generation AT using the categories of activities generated in phase one to create subject-tool-object activity system diagrams (see Figs. 1 and 2) for each student participant. This step served to make explicit what tools and artifacts mediated student participants’ goals to help describe how they engaged during the scenario. The use of third-generation AT supported analysis of scenarios in which multiple student participants were engaged (e.g., uncomplicated postpartum assessment) to support analysis of their interactions and multiple perspectives.

Following each stage of analysis, the findings were presented to an interpretive community for feedback and guidance. The interpretive community was comprised of two individuals with expertise in SBL in nursing education and two educational psychologists with expertise in studying complex learning environments. The community provided guidance related to categorizing participant activity and made recommendations about data analysis efforts.

## Results

### Activities student participants engaged in during scenario-based simulations

Three clinically relevant categories were identified, including (a) use of physical clinical tools and artifacts, (b) social interactions, and (c) performance of structured interventions. Table 2 summarizes these three categories of activities, their operational definitions, and examples from the data.

**Table 2:** Summary of clinically relevant activities, operational definitions, and identified examples

Clinically relevant activity	Operational definition	Examples
Use of physical clinical tools and artifacts	Physical items that are present in the simulated setting that form the simulated system that student participants may interact with or utilize to achieve their goals	Patient simulator, standardized participant, diagnostic tools (e.g., stethoscope), and diagnostic findings (e.g., lab results, vital signs)
Social interactions	Exchanges that student participants have with others in the simulated context, such as peers, standardized patients and other simulated participants (e.g., patient, patient’s support person, anesthesiologist). Social interactions are also considered tools that student participants interact with or use to achieve their goals.	Diagnostic questioning, education and counseling, social and emotional support, and situational management
Structured interventions	Activities that student participants perform that are governed by a set of predetermined rules guiding the processes of how or when they are used.	Diagnostic activities (e.g., auscultation, palpation), therapeutic interventions (e.g., medication administration), and patient safety practices (e.g., hand hygiene)

#### Physical clinical tools and artifacts

This category included physical items that formed the simulated system that student participants were expected to interact with or use to achieve their goals. Examples of physical clinical tools included a blood pressure cuff, stethoscope, thermometer, or personal protective equipment (e.g., alcohol gel, gloves). Clinical artifacts included diagnostic findings, such as vital sign data, physical assessment findings (e.g., fundal height, fetal waveforms, urine output), and lab results. Clinical artifacts were either provided to the participant as a component of the patient’s medical record or were derived when the participant engaged in an activity to determine the result. For example, the primary nurse in the uncomplicated postpartum assessment scenario was required to perform the assessment and determine fundal height.

#### Social interactions

This category included exchanges that student participants have with others in the simulated context, such as peers, standardized patients, and other simulated participants (e.g., patient, patient’s support person, and anesthesiologist). Social interactions are also considered tools because, according to AT, the use of language, often called a *sign*, is also deemed a tool that individuals may use to transform their environment. Social interactions were categorized into four sub-categories, including diagnostic questioning, education and counseling, social and emotional support, and situational management. Table 3 summarizes the categories of social interactions identified in all four ScBS, key characteristics, and examples from the scenarios.

**Table 3:** Summary of social interactions, key characteristics, and examples

Social interaction category	Key characteristics	Exemplar utterance
Diagnostic questioning	Interactions in which the student participant sought specific information from the patient, and/or their support person(s) to formulate a diagnosis, or assess the impact of a therapy	“How is your pain right now?”; “When was the last time you went to the bathroom?”
Education and counseling	Interactions in which the student participants tried to, a) prepare the patient for a future action, b) provide the patient or support person(s) with assessment findings, or c) instances where students explicitly provided patients with information about self-care.	“I’m going to take some vitals and check things out. OK?”; “I’m feeling that your uterus is hard and its shrunk down under your belly button which is great, that’s what we’re looking for right now.”; “You’ll probably get a little more cramping when you’re nursing.”
Social and emotional support	Interactions including statements intended to give reassurance, empathy, or encouragement. These interactions were directed to the patient or their support person(s).	“Yeah, those are both really natural questions to be wondering.”; “I know this is painful, but you can do this OK.”
Situational management	Interactions including statements where student participants sought to manage or direct patient care actions, such as seeking help or giving direction to peers assigned other roles. These interactions were directed towards peers or other healthcare professional roles.	“Hi, can I have nursery come in here?”; “I need some help in here.”; “I need someone to give ah…do massage and someone to get a straight cath.”

#### Structured interventions

These included activities that were governed by a set of predetermined rules or methods. For example, medication administration is guided by a specific series of rules that student participants follow to ensure that they are giving the correct drug, the correct dose, via the correct route, to the correct patient. Structured interventions also included activities such as palpation, auscultation, or visualization, during which the student participants used their hands, ears, and eyes in coordination with physical tools and props. For example, the auscultation of lung sounds required that student participants to use their stethoscopes and follow a pre-designated approach to obtain lung sounds. The complexity of structured interventions ranged from less complex to complex, including less complex interventions such as using an automated blood pressure device to assess blood pressure, to more complex, such as conducting a complete postpartum assessment.

## Frequency and regularity of activity

Among all four scenarios, regardless of scenario type, all three clinically relevant activities (i.e., physical clinical tools and artifacts, social interactions, and structured interventions) were present. Additionally, all student participants, regardless of role assignment or scenario goals, engaged in a combination of these activities. Table 4 provides a summary of the frequency of these activities among all student participants across all four scenarios.

**Table 4:** Frequency of participant clinically relevant activities in all four selected videos

Scenario name	Participant role	Use of physical clinical tools and artifacts	Social interactions	Structured interventions	Total activity/participant
Uncomplicated postpartum assessment					
	Primary nurse	18	*41*	9	68
	Support nurse	*20*	4	8	32
Post-epidural hypotension					
	Primary nurse	9	*25*	14	48
	Support nurse	*17*	16	4	37
Postpartum hemorrhage					
	Primary nurse	15	33	7	55
	Support nurse 1	*20*	7	1	28
	Support nurse 2	2	*31*	1	34
Fetal demise					
	Primary nurse	8	*76*	5	89

Italics indicate most frequent activity performed by participant

### How student participants engaged in scenario-based simulations

In addition to engaging in clinically relevant activities, there were three key themes related to *how* student participants engaged:

- Role assignment influenced student participants’ clinically relevant activities (i.e., use of physical clinical tools and artifacts, social interactions, structured interventions);
- Student participants sequenced and integrated clinically relevant activities, namely the use of physical clinical tools and artifacts, social interactions, and structured interventions, to make sense of the clinical presentation
- Student participants coordinated and distributed activity among peers assigned other roles.

Each of these themes is discussed below.

### Influences of assigned role

Role assignment influenced student participants’ combinations of activities. For example, across all four scenarios, student participants assigned the role of primary nurse engaged in more social interactions than student participants assigned as support nurse. This included social interactions with the patient, their support persons, and other healthcare professionals. Additionally, in all scenarios where multiple student participants engaged, primary nurses partook in more structured interventions than did student participants assigned as the support nurse. For example, in the uncomplicated postpartum assessment scenario where the goal was for student participants to complete an uncomplicated postpartum assessment, the primary nurse was responsible for conducting the complete assessment, whereas the support nurse’s activities were more focused, such as assessing vital signs or evaluating urine output (see Table 5). Furthermore, student participants assigned as the support nurse engaged in more uses of physical tools and artifacts while they completed their structured interventions. Additionally, student participants assigned to support nurse roles partook in fewer social interactions with the standardized patient, support persons, or other healthcare professionals.

**Table 5:** Summary of participant tool and artifact use by participant role

Primary scenario goal	Role	Physical clinical tools and artifacts	Structured interventions
Conduct an uncomplicated postpartum assessment	Primary nurse	Alcohol gel, gloves, vital sign monitor, stethoscope, disposable underwear, peri-pad	*Postpartum assessment*, apply alcohol gel, palpate, auscultate, visualize, assess blood loss (peri-pad), change peri-pad
	Support nurse	Alcohol gel, gloves, patient ID band, vital sign monitor, pulse oximetry monitor, thermometer, vital signs readings, heart rate, respiratory rate)	Apply alcohol gel, identify patient, assess vital signs (mother), assess blood oxygen, assess urine output, visualization, auscultation, hand washing
Escalate care and treat a patient experiencing post-epidural hypotension	Primary nurse	Alcohol gel, gloves, vital sign monitor, electronic fetal monitor (EFM), IV pump, thermometer, stethoscope	*Intrapartum assessment*, apply alcohol gel, auscultation, assess vital signs (mother), interpret EFM tracings, lower head of bed, roll patient, give fluid bolus
	Support nurse	Alcohol gel, gloves, electronic fetal monitor (EFM), pillow, IV pump	Apply alcohol gel, interpret EFM tracings, roll patient, fluid bolus
Recognize and treat a patient experiencing a postpartum hemorrhage	Primary nurse	Alcohol gel, gloves, Chux, vital sign monitor, pulse oximetry monitor, vital sign readings, straight catheter kit	*Postpartum assessment*, apply alcohol gel, assess vital signs (mother), assess blood oxygen, visualization, assess blood loss, perform straight catheterization
	Support nurse 1	Alcohol gel, gloves, Chux, IV fluids, IV tubing, IV pump	Fluid administration
	Support nurse 2	Alcohol gel, gloves	Fundal massage
Conduct an intrapartum assessment of a patient experiencing a fetal demise in utero	Primary nurse	Alcohol gel, gloves, vital sign monitor, pulse oximetry monitor, thermometer, vital sign readings, pulse oximetry reading, stethoscope	*Intrapartum assessment*, apply alcohol gel, apply gloves, assess vital signs, auscultate (lung sounds), assess blood oxygen

Italics indicate structured interventions with greater complexity

The analysis suggests this difference in activity may have occurred because student participants assigned the role of primary nurse were expected to complete more complex goals, such as conducting a complete uncomplicated postpartum assessment or recognizing that the laboring patient in the post-epidural hypotension scenario needed escalation of care. This added complexity is also reflected in the frequency counts, where in all four scenarios, primary nurses engaged in more activity overall (see Table 4). Additionally, in both the post-epidural hypotension and postpartum hemorrhage scenario, support nurses arrived later, and when they arrived, they were often assigned specific structured interventions, such as administering fluid or fundal massage, by the primary nurse. Table 5 provides a summary of the types of clinically relevant activities student participants engaged in in all four scenarios based upon their designated clinical role.

### Sequencing and integrating activities to make sense of the clinical presentation

*Student* participants’ observed performance of clinically relevant activities did not appear to be random; instead, they sought to thoughtfully sequence their activities to make sense of the clinical situation the scenario presented. Student participants sought to achieve the complex activity of sequencing by integrating clinically relevant activities (i.e., use of physical clinical tools and artifacts, social interactions, structured interventions). This included interactions with peers, the standardized patient, and simulated participants portraying the patient’s support person or other health care professional roles. For example, Table 6 presents a sequence of the activities and social interactions in which the participant portraying the role of primary nurse engaged while conducting a portion of the uncomplicated postpartum assessment.

**Table 6:** Making sense of the clinical presentation

Primary nurse	“OK, I’m just going to check your belly.” [Education and Counseling]
Primary nurse	Moves gown to expose abdomen.
Primary nurse	“Any discomfort in your belly or deep down in your abdomen?” [Diagnostic Questioning]
Patient	“Hmm…just a little cramping every now and then.”
Primary nurse	“OK, and you'll probably get a little more cramping when you’re nursing too.” [Education and Counseling]
Patient	“Yeah, it seems about when it is.”
Primary nurse	“Ok, I'm just going to give a quick listen to your belly.” “Have you been having any gas or anything moving?” [Diagnostic Questioning]
Patient	“Yeah, I feel it a little bit.”
Primary nurse	“OK...we just like to make sure everything is moving again.” [Education and Counseling]
Patient	“Yeah…okay.”
Primary nurse	Auscultates abdomen [Structured Intervention]
Primary nurse	Moves sheets down, places left hand at the base of the patient's’ belly and with right hand palpates for the top of the fundus. [Structured Intervention]
Primary nurse	“OK, I’m just gonna feel, see if I can feel the placement of your uterus [Goal], to see if it's shrinking down again.”
Primary nurse	Palpates fundus. [Structured Intervention]
Primary nurse	“And right here, I'm going to document this as 2 under.” [Education and Counseling]
Patient	“Hmm, it’s a little sore when your push.”
Primary nurse	“Okay, sorry about that. [Social and emotional support] But, what I’m feeling is good. I’m feeling that your uterus is hard and it shrunk down under your belly button, which is great, that's what we're looking for right now. Every time you have those cramps, is because your uterus is is..uh, getting back to its normal shape.” [Education and Counseling]

The student participant portraying the primary nurse appears to thoughtfully sequence her structured interventions and social interactions (including gathering information from the patient) to construct an understanding of the patient’s condition and uterine height. She makes her understanding of the situation explicit when we she educates and counsels the patient on her findings

In this example, the primary nurse sequenced her activities by first educating the patient about her assessment plans, performing the planned structured intervention of auscultating the abdomen, followed by palpating for the height of the fundus. Additionally, together with her physical exam findings, the primary nurse further made sense of the patient’s clinical presentation by engaging in dialog with the patient by asking additional diagnostic questions. This example highlights how the use of physical tools and artifacts *and* social interactions are critical to the sense-making process. Additionally, she made her understanding of the findings explicit when she verbalized her documentation plans and when she educated and counseled the patient about her findings. Student participants in all four scenarios demonstrated similar patterns of activity.

Efforts to sequence clinically relevant activity included instances in which student participants were successful (e.g., Table 6) as well as instances in which they struggled. Table 7 provides a selected sequence of activities conducted by the primary and support nurses during the post-epidural hypotension scenario. In this scenario, the primary and support nurses initially struggle to coordinate their efforts to provide care when the primary nurse relays limited information about the patient’s situation. Prior to the sequence in Table 7, the primary nurse had started her intrapartum assessment, determined that the patient was experiencing hypotension, lowered the head of the bed, called for help, and partially rolled the patient on her side.

**Table 7:** Struggling to make sense and coordinate

Primary nurse	“Ok, so I think that is kind of a reaction from the epidural [Education and Counseling], we just want to make sure we increase your fluids” [Goal]
	“I’m going to hit the call button and get a second nurse in here.” [Goal]
Primary nurse	Presses call button [Clinical Tool]
Support nurse	“Hi, how's it going? What's going on?” [Situational Management]
Primary nurse	“First, if you could get some extra pillows to turn her on her side, and then call the anesthesiologist back.” [Situational Management]
Support nurse	Observes fetal tracing and maternal vital signs [Clinical Artifacts]
Support nurse	“So, you just got an epidural?” [Diagnostic Question]
Patient	“Yeah, uh..is there something wrong?”
Support nurse	“So sometimes when we give an epidural we can have your blood pressure drop down a little bit, so were going to try and um, get that kind of…back up a little bit [Goal]. So were going to roll you over on your left side a little bit more.” [Education/Counseling]
Support nurse	(to primary nurse) “Do you want to…uh…roll her over a bit more?” [Situational Management]
Primary nurse	“…Uh, yeah…Okay.”
Support nurse	(to primary nurse) “*I'm just going to get another pillow so we can get her all the way over on her left.”* [Situational Management]
Support nurse	Exits and re-enters the room with an additional pillow [Clinical Tool]

The two student participants portrayed in this scenario struggle to coordinate their care. Although the primary nurse appears to have some understanding of the clinical presentation, she struggles to describe the situation to the support nurse when she arrives. Faced with this, the support nurse makes a quick assessment of the situation by examining the fetal heart rate tracing and the maternal vital signs. She makes her understanding of the situation explicit when she describes the situation to the patient and then again when she recommends a plan to the primary nurse to roll the patient on her side

In this example, the participant portraying the primary nurse struggled to provide a cohesive accounting of the patient’s situation to the support nurse. This interaction, in turn, led the support nurse to quickly assess the situation herself by conversing with the patient and evaluating the patient’s vital signs (clinical artifact). Although this exchange highlights how a student participant can struggle to engage in ideal sequencing of activities, the student participants in this vignette still made the effort to thoughtfully sequence their care.

### Coordination and distribution of activity

Student participants achieved their goals by making sense of the clinical presentations by coordinating their efforts with their peers assigned supportive clinical roles, the standardized patient and simulated participants portraying other roles (e.g., charge nurse). Coordination of activity with others was present in all four scenarios and included efforts to perform structured interventions and meet the faculty-intended goals of the scenarios. For instance, the previous example in Table 7 demonstrates how the support nurse’s interactions buttressed and guided the primary nurse’s activity. Additionally, in the fetal demise scenario, although the participant engaged in the scenario without the co-presence of another nursing student or healthcare professional, she coordinated her activity with the patient and her support person. This highlights the critical role that all participants in a ScBS (e.g., student participants, standardized patient(s), and simulated participants) can play.

In addition to coordinating efforts, student participants also distributed the workload of care across multiple team members. For example, in the uncomplicated postpartum scenario, the primary nurse and secondary nurse coordinated their activities to complete the goal of an uncomplicated postpartum assessment (see Table 5). Together, they achieved the appropriate provision of care. Figure 2 highlights how three student participants collaborated and distributed care to support a patient experiencing a postpartum hemorrhage.

The activity diagrams in Fig. 2 highlight how student participants distributed the workload across three nursing students to obtain the shared goal of stopping the postpartum hemorrhage in a hemodynamically unstable patient. Although all student participants worked towards the same goal, each undertook a specific goal (e.g., catheterizing the patient, administering fluids).

## Discussion

The findings derived from analyzing participant activity demonstrate the multiple ways in which ScBSs can support learning: engagement in clinically relevant activities (i.e., use of tools, social interactions, and structured interventions), sequencing clinically relevant activities and social interactions to make sense of the clinical presentation, and coordination and distribution of the workload. The frequency and regularity analysis shows that these activities were consistent among all participant roles and across all four scenarios. The activity and frequency analysis also suggests that role assignment can influence the complexity of student participants’ experiences. For example, Table 5 demonstrates how student participants assigned the role of primary nurse conducted more complex tasks or had greater levels of responsibility as compared to support nurses.

Importantly, the findings suggest that these scenarios afforded student participants the opportunity to reflexively sequence clinically relevant activities, which includes their practice of aggregating patient data and diagnostic artifacts, to make sense of the patient’s clinical presentation and make decisions related to treatment and care. Students’ activities also included their reflexive interactions with other student participants, standardized patients, and other simulated participants (e.g., charge nurse), further highlighting the complex activity systems created when working with ScBSs. Moreover, when a scenario required more than a single student participant to provide care, they coordinated their activities with each other and distributed the workload to achieve their goals. Thus, in addition to the individual performance of clinically relevant activities, engagement with standardized patients and other simulated participants and peers was an integral component of sense-making efforts during scenario participation.

### Implications for instructional design features of scenario-based simulations

These findings are consistent with prior research emphasizing the role that simulations play in affording student participants access to practice diverse, clinically related skills [2, 10]. The findings are also consistent with prior guidance indicating that scenarios support learning to interact with conscious patients and other team members [3, 5]. Importantly, by focusing on participant activity, these findings *make explicit* some of the major categories of activities student participants may gain access to during ScBS participation, specifically the use of physical tools and artifacts, diverse social interactions, and structured interventions.

The findings from the activity analysis indicate that student participants did not partake in focused repeated practice of a specific clinically relevant activity during a single scenario. However, when considering student participants’ activity across all four scenarios (see Table 5), the data showed that opportunities to repeat practice of some activities were distributed across multiple scenarios (e.g., postpartum or intrapartum assessment, interpretation of vital signs). Thus, these findings extend Issenberg et al.’s and McGaghie et al.’s reviews by shedding light on how educators could *operationalize* repeated practice opportunities when employing ScBS in their curricula [1, 2].

The frequency and regularity analyses may offer new insights into ways to consider scaffolding student participants’ experiences by thoughtfully considering the complexity of each role portrayed in the scenario. For example, the analysis suggests that student participants assigned to roles with greater levels of responsibility (i.e., primary nurse) conducted more complex care, relied on more subjective artifacts (e.g., palpation of the fundus), and were required to determine when and what support persons were assigned to do. Thus, simulation stakeholders could consider assigning student participants with greater amounts of training or ability to more complex roles. Conversely, less experienced student participants could be considered for support roles that may entail less complexity. This may enable learners of diverse abilities to simultaneously partake in a ScBS.

Considering this scaffolding approach is consistent with Lave and Wenger’s theories regarding legitimate peripheral participation (LPP). LPP indicates that newcomers, such as novice student participants in a scenario, can gain greater experience in a community of practice when they have access to opportunities to engage in simple or lower risk tasks that are nonetheless important to the community’s goals [28]. Furthermore, per Lave and Wenger, participants benefit from both direct participation in a meaningful activity, while also benefiting from modeling provided by their more capable peers [28]. Additionally, scaffolding using such an approach is consistent with recent best practice guidance issued by the NCSBN, which indicate that simulation objectives should be aligned with student participants’ developmental level [29].

Furthermore, the findings are also consistent with prior guidance associated with selecting scenarios to give student participants opportunities to practice diverse communication skills [5], breaking bad news, [30] or supporting clinical situations related to death and dying [31]. Importantly, the activity systems analysis highlights how ScBS affords student participants access to selecting and sequencing social interactions with clinically relevant activities (see, for example, Tables 6 and 7). These findings may provide insight into how partaking in ScBS might support learning clinical reasoning or engage in diagnostic decision-making.

### Implications for learning in scenario-based simulations

The findings are also similar to results from Kneebone et al., Lasater, and Mikkelsen et al., who reported that student participants experienced learning during ScBS as occurring through engagement in ScBSs [3, 15, 16]. The descriptive use of AT revealed how student participants transformed objects and how other system components, such as social interactions with peers and standardized patients or accessing and interpreting diagnostic findings, mediated this transformation. Although previous literature has emphasized reflection and debriefing as the primary ways to engage in sense-making associated with ScBS [12–14], the findings of this study suggest that sense-making may take place during ScBS participation as well.

By undertaking an in-depth analysis of participant activity, the findings also highlight the complex and emergent properties of these ScBS activity systems. This was exemplified in the activity analysis in which the components (e.g., subject, tools, objects) of the ScBS activity system were not isolated from each other but were dynamic and continuously interacting with each other (see Tables 6 and 7, Fig. 2 for example) [17, 18, 32].

The activity analysis also highlights the *nested activities* within the ScBS activity system [32]. Barab and colleagues define nested activities as those activities or actions that could be conceived of as separate activity systems. For example, although the faculty-selected goals of these scenarios were explicit, the students often voiced their own goals during the scenario (see, Fig. 2, for example). This nestedness was especially highlighted in the team-based scenario in which each participant’s activities differed, such as one participant’s practice of external uterine massage while another participant prepared medication, yet they all worked towards the common goal of resolving the postpartum hemorrhage. These complex, emergent, and nested properties could have significant implications for formative and summative assessment of student participants.

Lastly, the findings potentially extend our understanding of how scenarios may support learning team-work behaviors when they afford student participants opportunities to coordinate care and distribute the workload with peers and other healthcare professionals, while simultaneously interacting with the material environment (e.g., stethoscope, thermometer, diagnostic findings). These characteristics are consistent with Hutchins’ [33] concept of distributed cognition which suggests that interaction is “deeply multimodal and composed of a complex network of relationships” (p. 376) [33]. Multimodality refers to the different embodied mediums or tools (e.g., physical tools, social interactions) that individuals use to achieve their goals [33]. This complexity and multimodality is reflected in Tables 4, 5, 6, and 7 and in Fig. 2, which highlight the frequency and diversity of activities in which student participants engaged. Thus, ScBS could alternatively be framed as *simulated clinical systems* in which the unit of analysis would include examination of how student participants coordinate while integrating the use of culturally relevant artifacts to achieve a goal. Viewing scenarios as simulated clinical systems potentially provides simulation stakeholders with alternative ways to examine how student participants collectively achieve goals that go beyond solely relying on verbal reflection or outcomes assessment of performance [34].

### Limitations

Although this study provides an in-depth examination of the types of activities student participants engaged in during high-quality ScBS participation, the strategy of rich description required the use of a limited number of video-recorded scenarios. Additionally, these videos depicted senior nursing student participants who could function independently with limited or no support from faculty, thus the analysis may only reveal the types of activities in which more experienced student participants engage. Future research should include analysis of diverse levels of learners (e.g., novice, intermediate) and diverse types of learners (e.g., physicians, respiratory therapists) who partake in SBL activities. Furthermore, the choice to use diverse types of scenarios did not allow for analysis of how consistent the patterns of activity were for a single scenario type (e.g., communication). Future research should take consistency into consideration. Lastly, although this analysis provides a framework that can be used to describe how observing student participants’ activities may support learning, this analysis did not include participants’ reflections on their activity. Future efforts could include the use of stimulated video-recall, which could be used to triangulate student participants’ intended goals.

## Conclusions

This study makes explicit the types of activities in which student participants across diverse types of ScBSs engage, which offers important insight into what student participants practice during such an activity. The findings also suggest that learning within scenarios may take place while student participants work to sequence their activities and make sense of the clinical presentation; in this way, the findings add an alternative perspective to how ScBSs may support learning. Importantly, these findings add new insights into the nuances and complexity associated with scenario participation and offer added detail about how scenarios can be designed and implemented to scaffold student participants’ learning.

The findings also raise new questions about how simulations support learning and simulation-based instructional design practices; these findings warrant further investigation. Questions may include the following: (a) are these activity codes consistent among diverse health professions disciplines (e.g., physicians, nursing, allied health professionals), areas of specialization (e.g., emergency medicine, anesthesia), and a continuum of participants (e.g., students, practitioners, interprofessional teams); (b) how might activities in skills-based or procedurally based simulations be similar or different; (c) how do student participants’ activities change or differ as they develop or gain greater understanding over time; (d) how do standardized and simulated participants’ activities contribute to the learning process in a ScBS; and (e) how might embodied communication (e.g., gesture, visual gaze) contribute to sense-making, coordination and distribution of activity in a ScBS?

## Abbreviations

AT: Activity theory
BEME: Best Evidence Medical Evaluation
DP: Deliberate practice
IRB: Institutional review board
LPP: Legitimate peripheral participation
NCSBN: National Council of State Boards of Nursing
SBL: Simulation-based learning
SBME: Simulation-based medical education
ScBS: Scenario-based simulation
